# The Roles of Bone Marrow-Derived Stem Cells in Coronary Collateral Growth Induced by Repetitive Ischemia

**DOI:** 10.3390/cells12020242

**Published:** 2023-01-06

**Authors:** Molly Enrick, Anurag Jamaiyar, Vahagn Ohanyan, Cody Juguilon, Christopher Kolz, Xin Shi, Danielle Janota, Weiguo Wan, Devan Richardson, Kelly Stevanov, Tatevik Hakobyan, Lindsay Shockling, Arianna Diaz, Sharon Usip, Feng Dong, Ping Zhang, William M. Chilian, Liya Yin

**Affiliations:** 1 Department of Integrative Medical Sciences, Northeast Ohio Medical University, Rootstown, OH 44272, USA; 2 Department of Anatomy and Neuroscience, Northeast Ohio Medical University, Rootstown, OH 44272, USA

**Keywords:** bone marrow stem cells, coronary collateral growth, repetitive ischemia, coronary blood flow, coronary microcirculation

## Abstract

Many clinical trials have attempted to use stem cells to treat ischemic heart diseases (IHD), but the benefits have been modest. Though coronary collaterals can be a “natural bypass” for IHD patients, the regulation of coronary collateral growth (CCG) and the role of endogenous stem cells in CCG are not fully understood. In this study, we used a bone marrow transplantation scheme to study the role of bone marrow stem cells (BMSCs) in a rat model of CCG. Transgenic GFP rats were used to trace BMSCs after transplantation; GFP bone marrow was harvested or sorted for bone marrow transplantation. After recovering from transplantation, the recipient rats underwent 10 days of repetitive ischemia (RI), with echocardiography before and after RI, to measure cardiac function and myocardial blood flow. At the end of RI, the rats were sacrificed for the collection of bone marrow for flow cytometry or heart tissue for imaging analysis. Our study shows that upon RI stimulation, BMSCs homed to the recipient rat hearts’ collateral-dependent zone (CZ), proliferated, differentiated into endothelial cells, and engrafted in the vascular wall for collateral growth. These RI-induced collaterals improved coronary blood flow and cardiac function in the recipients’ hearts during ischemia. Depletion of donor CD34^+^ BMSCs led to impaired CCG in the recipient rats, indicating that this cell population is essential to the process. Overall, these results show that BMSCs contribute to CCG and suggest that regulation of the function of BMSCs to promote CCG might be a potential therapeutic approach for IHD.

## 1. Introduction

Ischemic heart diseases (IHD) are the leading cause of mortality and morbidity in the United States. Various studies of cell-based regenerative therapy in myocardial infarction and many trials have examined the effects of stem cells on ischemic heart disease (refer to clinicaltrials.gov, accessed on 3 January 2023), but the outcomes are mixed [1,2]. Although some efforts have been directed towards the stimulation of collaterals in peripheral arterial disease, very few of these trials are aimed explicitly at stimulating coronary collateral growth (CCG) in IHD. Coronary collaterals are anastomoses between two adjacent branches of the coronary arterial tree, allowing blood to bypass the atherosclerotic lesion and supply the under-perfused myocardium downstream of the stenosis, restoring oxygen supply [3]. Stimulating collateral growth in the heart can prevent myocardial infarction or sudden death. It is also an alternative therapeutic approach for patients with intractable angina pectoris who are not indicated for percutaneous coronary intervention and/or coronary artery bypass grafting [4,5,6]. Importantly, patients with well-developed coronary collaterals have a better prognosis in recovering from a myocardial infarction than those with poorly developed collaterals [7]. We believe stimulation of CCG might be an opportunity for regenerative therapies to work in a shorter period in treating IHD. However, the postnatal process of CCG is not entirely understood [8]. Before adopting any stem cell therapy to induce CCG, it is critical to investigate whether endogenous bone marrow stem cells (BMSCs) contribute to postnatal CCG. In this study, we will address this question through bone marrow transplantation in a rat model of CCG. Furthermore, we will investigate which cell type(s) in the bone marrow is essential to CCG. The results from this study show that BMSCs contribute to CCG induced by repetitive ischemia, indicating a potential therapeutic approach to induce CCG in IHD by regulating BMSC function.

## 2. Materials and Methods

### 2.1. Animals

All procedures were conducted with the approval of the Institutional Animal Care and Use Committee of Northeast Ohio Medical University and following National Institutes of Health Guidelines for the Care and Use of Laboratory Animals (NIH publication no. 85–23, revised 1996). Sprague–Dawley (SD) rats were purchased from Charles River Laboratories (Wilmington, MA, USA), and hemizygous SD-Tg (UBC-EGFP) 2BalRrrc rats were purchased from the Rat Resource & Research Center (Columbia, MO, USA). Rats were housed in a temperature-controlled room with a 12:12-h light–dark cycle and maintained with access to food and water ad libitum.

### 2.2. Bone Marrow Collection

Bone marrow was harvested from adult male rats, either SD rats or hemizygous transgenic SD rats expressing ubiquitous green fluorescent protein (GFP). The rat femurs and tibias were flushed with 50% FBS in PBS using a 21G needle, and the collected bone marrow cells were pelleted by centrifugation. The red blood cells (RBC) were lysed with RBC lysis buffer diluted from 10× RBC Lysis Buffer (Multi-species) (eBioscience, # 00-4300-54), the bone marrow cells were washed with PBS, and the cell numbers were counted and used for staining or transplantation.

### 2.3. Bone Marrow Transplantation

Recipient SD rats were administered acidified drinking water (pH = 2.5–3.0) supplemented with neomycin (150 mg/L) and bacitracin (5 mg/L) for at least one-week pre-transplant and 4 weeks post-transplant. On the day of the transplant, recipient rats were irradiated using an X-ray irradiator (RS2000, Rad Source Technologies, Suwanee, GA, USA) with 2 doses of 5 Gy given 4 h apart. They were then anesthetized with 1–3% isoflurane via a nose cone, and a lateral femoral incision was made. More than 2 × 10^7^ cells were injected intraosseously in each femur with a 23 G needle. Rats were allowed to recover for around 4–6 weeks until hematocrit and hemoglobin levels recovered. Blood was collected from the tail vein in anesthetized rats under 1–3% isoflurane or from the inferior vena cava at the time of sacrifice. Whole blood was collected in a lavender-top EDTA tube and shipped overnight on ice to VRL Maryland (Gaithersburg, MD, USA) for hematology analysis.

### 2.4. Flow Cytometry and Sorting

Bone marrow cells were incubated with 5% BSA for 30 min and stained at 10^7^/µL with PE Anti-CD34 (ab223930) or isotype control (ab209478) for 1 h at 4 °C. The cells were then washed and sorted for CD34^+^ and CD34^−^ cells by BD FACSDiva or BD FACS Aria Fusion System. Alexa Fluor647 conjugated sc-168 c-kit Antibody (C-19) and isotype control were used for staining c-kit^+^ cells. Cells were stained, washed, and fixed in 1% PFA for flow cytometry analysis with C6 Flow Cytometer (BD Biosciences).

### 2.5. Rat Model of Coronary Collateral Growth Induced by Repetitive Ischemia (RI)

BMSC recipient rats were used for chronic implantation of a pneumatic occluder over the left anterior descending coronary artery (LAD), as described before [9]. Briefly, the rats were anesthetized and intubated, and the left chest was opened between the 3rd–5th intercostal spaces. An occluder was attached to the left ventricle over the LAD; the chest was closed, and the occluder tubing was exteriorized. Rats were allowed to rest for 3 days before undergoing the RI protocol for 10 days. During RI, short episodes of inflation of the occluder caused periodic ischemia in the collateral-dependent zone, downstream of the occluder but no infarction. The extent of ischemia depends on the abundance of coronary collaterals.

### 2.6. Echocardiographic Analysis of Cardiac Function

In vivo heart function was evaluated by M-mode echocardiography. All measurements were performed before and after inflation of the occluder on day 0 and day 10 of the RI protocol [9]. Percentage changes of left ventricle (LV) ejection fraction (EF) before and after inflation of the occluder will show the abundance of coronary collaterals; the difference in the percentage change between day 0 and day 10 will show the growth of coronary collaterals.

### 2.7. Measurements of Myocardial Blood Flow

Coronary collateral growth was evaluated from blood flow to the collateral-dependent region during the inflation of the occluder. We used two methods to measure the blood flow in the myocardium. In the first, neutron-activated microspheres were injected into the LV lumen during inflation of the occluder, then the myocardial tissue was collected for measurements [9]. The collateral flow was calculated as a ratio between the activity (dpm/g) of the tissues from the collateral-dependent zone and the normal zone (CZ/NZ) [9]. The second method is to measure the myocardial blood flow by contrast echocardiography with a Sequoia 512 (Siemens Medical Systems) before and during inflation of the occluder [10]. The mice were infused with contrast reagent (microbubbles; 20 µL/min, 5 × 105 bubbles/min) via the tail vein. Long axis images of the LV were obtained during a high-energy pulse sequence that destroys microbubbles and for several seconds after destruction to establish refilling of the chamber and ventricular wall. The collateral flow was calculated as the ratio of myocardial blood flow from the collateral-dependent zone and the normal zone (CZ/NZ) [8]. In both methods, the flow in the CZ and NZ should be roughly equal when the occluder is deflated, giving a CZ/NZ ratio of one. Theoretically, if there are no collaterals in the CZ, when the occluder is inflated, the ratio of CZ/NZ should be zero, reflecting the ischemia in the CZ. If the number of collaterals is enough to compensate for the deficiency of flow caused by inflation of the occluder, which temporarily cuts off the blood flow through LAD, the ratio of CZ/NZ should be one, even with inflation.

### 2.8. Immunostaining and Imaging

Rat hearts were sliced for multi-photon imaging or embedded, sectioned, and stained for confocal imaging [8,11]. The frozen heart sections were fixed in cold acetone, washed in PBS, blocked with blocking buffer, and stained with primary and/or secondary antibodies for imaging. Before sacrificing, some rats were perfused with Alexa Fluor 594 Conjugate Isolectin GS-IB_4_ (#I21413). To label proliferating cells, EdU (5-ethynyl-2′-deoxyuridine) from ThermoFisher was injected intraperitoneally in rats at 50 mg/kg body weight every 8 h for 3 days. Frozen heart sections were stained using a Click-iT EdU Cell Proliferation Kit for Imaging as described previously [8].

### 2.9. Retrograde Microfil Perfusion of Rat Hearts and Micro-Computed Tomographic (Micro-CT) Analysis

Rat hearts were perfused with Microfil polymer until the coronary arterial circuit was completed. The excised hearts were cleared and scanned using a Skyscan 1172 micro-CT scanner (Bruker) and reconstructed using Avizo software (Thermo Fisher Scientific, Waltham, MA, USA) for detection of collaterals and vascular tree analysis [8].

### 2.10. Statistics

Data are presented as mean ± standard deviation (SD). Data were analyzed using GraphPad Prism 7.0 statistical software. Comparisons between the two groups were made using an unpaired Student’s *t*-test. The parametric test was used when the normal distribution test showed that the data distributions did not significantly differ from normal; otherwise, a nonparametric test was used. Differences were considered statistically significant at a value of *p* < 0.05.

## 3. Results

### 3.1. Bone Marrow Stem Cells (BMSCs) Rescued the Recipient Rats from Lethal Irradiation after Transplantation and Repopulated the Chimeras’ Bone Marrow

**Figure 1:**
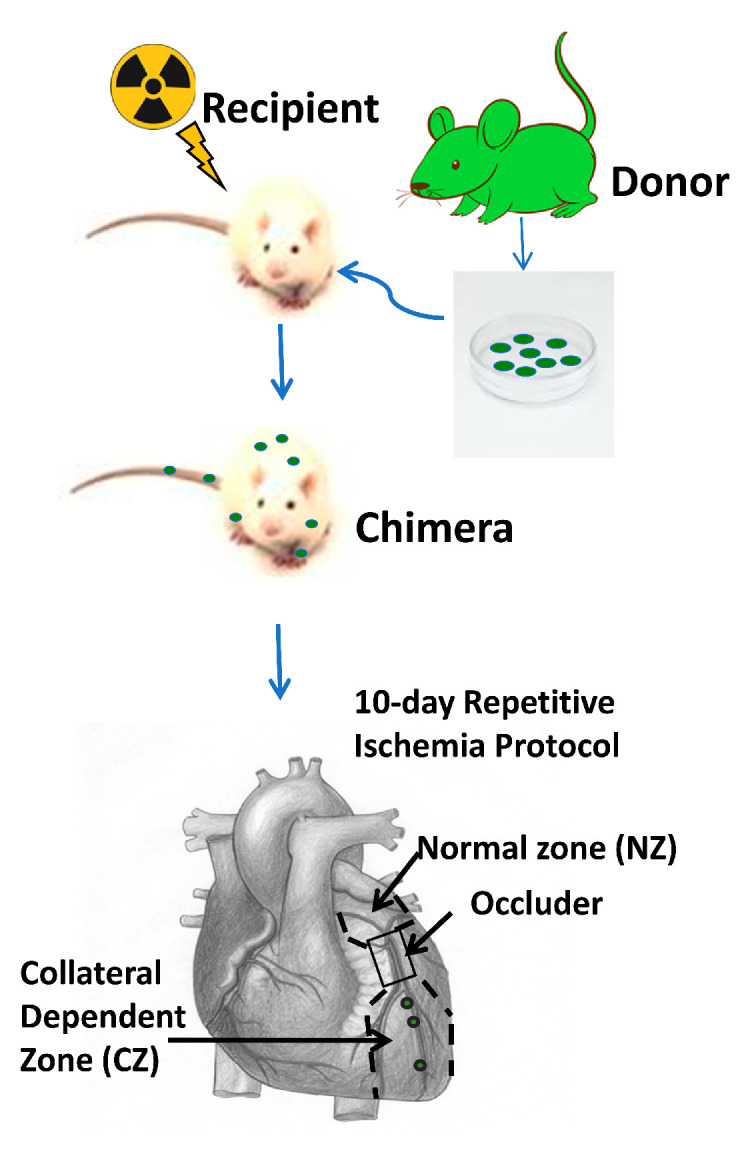
Schematic of bone marrow transplantation and rat coronary collateral growth (CCG) model induced by repetitive ischemia (RI). RI protocol was to occlude LAD for 40 s every 20 min for 3 h, then rest 5 h. Repeat for 10 days. Figure designed using assets from Freepik.com.

**Figure 2:**
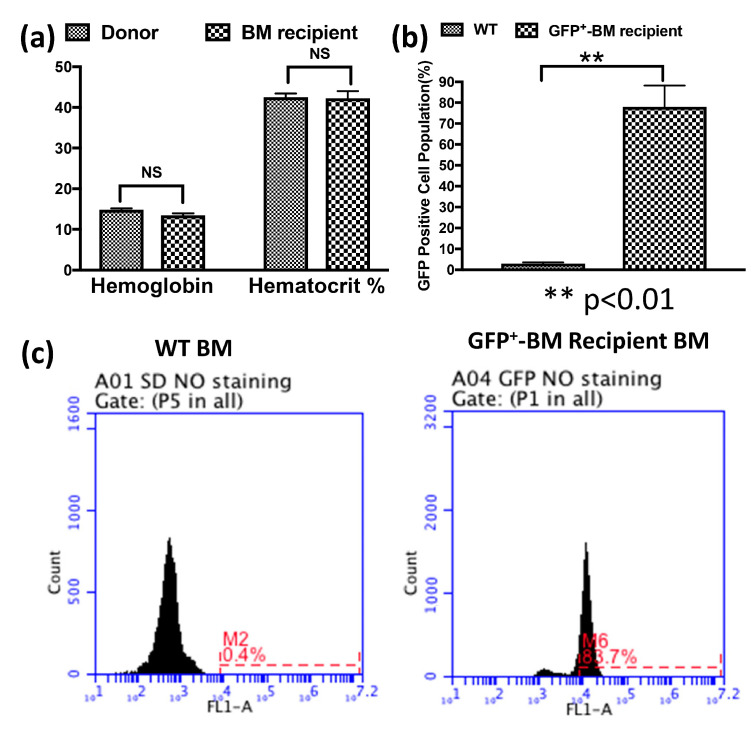
Transplanted bone marrow (BM) cells repopulated the blood cells of irradiated recipient rats. (**a**) There is no significant difference in hemoglobin or hematocrit between donor and chimeric rats receiving BM transplantation. (**b**) Around 80% of BM cells in the rats that received GFP^+^ BM and underwent RI are GFP positive. (**c**) shows the flow cytometry analysis of the bone marrow from WT SD rats and the chimeric SD rats that received GFP^+^ BM.

To study the role of BMSCs in coronary collateral growth (CCG), we adopted the bone marrow transplantation model shown in Figure 1. First, we optimized the dosage and interval of irradiation so that the rats receiving a lethal dose died without receiving bone marrow transplantation. Second, we checked the time needed for the chimeras to recover from the irradiation. Blood samples from the chimeras were sent out for hematology analysis 4–6 weeks after bone marrow transplantation. Figure 2a shows no significant difference in the hemoglobin and hematocrit levels between the chimeras and the donor rats, suggesting donor BMSCs repopulated recipient rats’ deficient circulating blood cells. To trace the fate of donor BMSCs after transplantation, we used donor rats expressing GFP. We analyzed the population of bone marrow cells that were GFP positive (GFP^+^) in the recipient rats by flow cytometry. Figure 2a,b shows that in the rats that received GFP^+^ BM and underwent RI, around 80% of bone marrow cells were GFP positive, further confirming that the donor bone marrow repopulated the bone marrow of recipient rats. These results are consistent with the literature, and we can further study the role of these transplanted BMSCs in CCG in our rat model [9].

### 3.2. GFP^+^ BMSCs Engrafted in the Collateral-Dependent Zone of Chimeric Rat Hearts and Differentiated into Endothelial Cells

**Figure 3:**
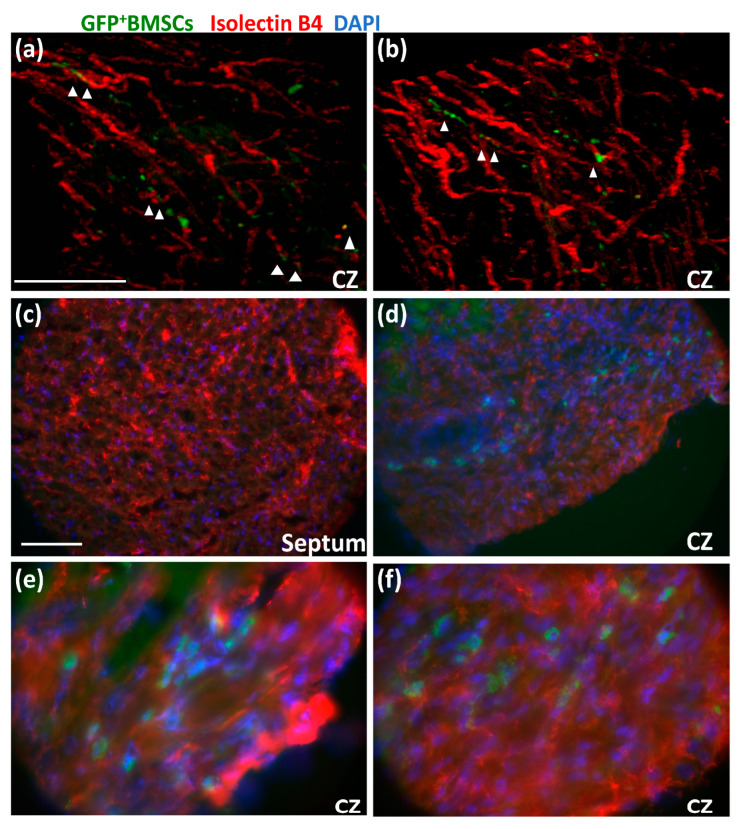
Bone marrow-derived stem cells (BMSCs) engrafted in blood vessels in the collateral dependent zone (CZ) of chimeric rats with GFP^+^ BM during repetitive ischemia (RI) protocol. (**a**,**b**). Multi-photon images of CZ of chimeric rat heart. Arrowheads indicate the colocalization of GFP ^+^ BMSCs with EC marker isolectin-B4. The scale bar equals 100 μm. (**c**,**d**). Confocal images of sections of the septum (**c**) and CZ (**d**) of chimeric rat heart. The scale bar equals 100 μm. (**e**,**f**) are high magnification images of (**d**), showing that GFP^+^ BMSCs were recruited to the CZ and differentiated into endothelial cells (EC) during coronary collateral growth induced by RI. Green shows GFP^+^ BMSCs. Red indicates the EC marker (isolectin-B4). Blue shows nuclei.

After the chimeric rats recovered, we performed surgery to implant an occluder over the LAD, and the rats underwent a 10-day repetitive ischemia (RI) protocol, as shown in Figure 1. We defined two zones in the left ventricle: the area above the occluder is designated as the normal zone (NZ), and the area below the occluder is designated as the collateral-dependent zone (CZ), which is also called the ischemia zone (IZ) because this area will be ischemic during inflation of the occluder, and the extent of coronary blood flow is dependent on the abundance of collaterals in this area. After the RI protocol, the chimeric rat cardiac tissues were harvested for imaging. First, we looked for GFP^+^ BMSCs in the chimeric rat heart tissue slices through a multi-photon microscope. Before sacrifice, the rats were perfused with Alexa-594-Isolectin-B4, an endothelial cell (EC) marker. Figure 3a,b shows that GFP^+^ BMSCs in green are present in the CZ. Interestingly, some green cells colocalized with endothelial cells shown in red, suggesting transplanted GFP^+^ BMSCs migrated from the bone marrow to the CZ of the myocardium and engrafted in blood vessels under the stimulation of RI.

To confirm whether the GFP^+^ BMSCs differentiated into EC, as suggested by these multi-photon images, we sectioned the heart tissues and imaged the sections with a confocal microscope. Figure 3c shows that almost no GFP^+^ BMSCs are presented in the septum of the chimeric rat heart. However, Figure 3d shows that many GFP^+^ BMSCs presented in the CZ were colocalized with red isolectin-B4, suggesting that GFP^+^ BMSCs differentiated into EC. Figure 3e,f shows high-magnification images of Figure 3d. These results indicate that GFP^+^ BMSCs were recruited to the CZ during the RI protocol, differentiated into EC, and contributed to CCG.

### 3.3. Engrafted GFP^+^ BMSCs Proliferated during RI

**Figure 4:**
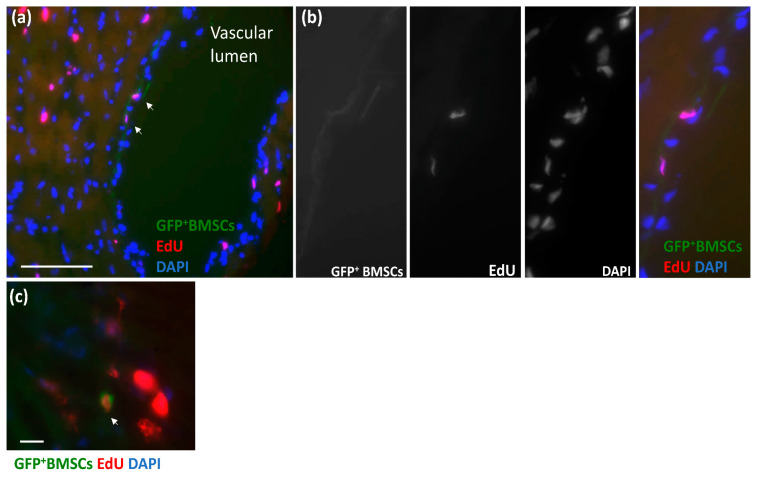
GFP^+^ BMSCs proliferated in the chimeric rat heart during coronary collateral growth induced by repetitive ischemia. (**a**) GFP^+^ BMSCs aligned in the vascular wall of the vascular lumen, co-localizing with 5-ethynyl-2’-deoxyuridine (EdU), which labels the proliferated cells. The scale bar equals 100 μm. (**b**) High-magnification images of the area with arrows. Green shows GFP^+^ BMSCs. Red shows proliferated cells labeled by EdU. Blue shows nuclei. (**c**) Another image of GFP^+^ BMSCs co-localizing with EdU. The scale bar equals 10 μm. Green shows GFP^+^ BMSCs. Red shows proliferated cells labeled by EdU. Blue shows nuclei.

To examine whether the GFP^+^ BMSCs homing to the CZ during RI proliferated and contributed to CCG, we injected 5-ethynyl-2′-deoxyuridine (EdU), which labels proliferated cells at day 3–5 of the RI protocol. Then the tissue sections of the CZ were stained with Click-iT EdU Cell Proliferation Kit. Figure 4a shows that GFP^+^ BMSCs aligned in the vascular wall of a vascular lumen, co-localizing with EdU. Figure 4b shows high-magnification images of the area with arrows from Figure 4a. Figure 4c shows another picture of GFP^+^ BMSCs co-localizing with EdU. These results indicate that GFP^+^ BMSCs recruited to the CZ were proliferated and engrafted to the blood vessels.

### 3.4. More c-kit^+^GFP^+^ BMSCs Homed for CCG during RI

**Figure 5:**
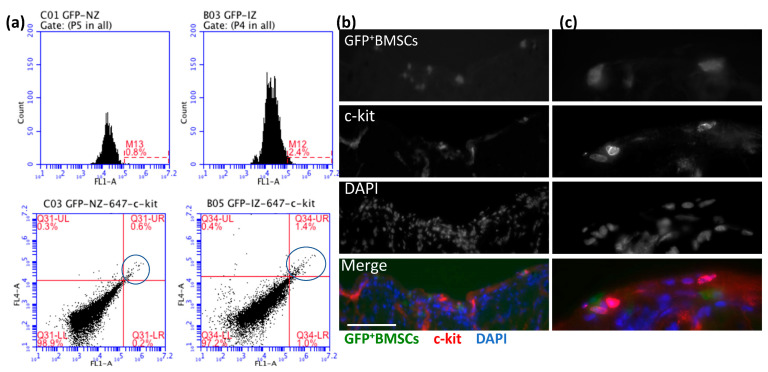
GFP^+^ or GFP^+^ c-kit^+^ bone marrow-derived stem cells (BMSCs) presented in the normal zone (NZ) and ischemia zone (IZ)/collateral dependent zone (CZ) of chimeric rat hearts. (**a**) Flow cytometric analyses of the heart of GFP^+^BM chimera after 10-day RI. Left: NZ GFP^+^ cells are 0.8% and c-kit^+^ 0.6%. Right: IZ GFP^+^ cells are 2.4%, and c-kit^+^ cells are 1.4%. Blue circles denote c-kit+ fractions. (**b**) Transplanted GFP^+^ BMSCs engrafted in the CZ of a chimeric rat heart expressed c-kit imaged by confocal microscopy. Green shows GFP^+^ BMSCs. Red shows c-kit^+^ cells. Blue shows nuclei. The scale bar equals 100 μm. (**c**) High magnification images of (**b**).

To better characterize the GFP^+^ BMSCs present in the CZ during RI, we digested the heart tissues from CZ (IZ) and NZ and checked the cell markers of GFP^+^ BMSCs by flow cytometry. One population of interest is c-kit^+^ BMSCs, which are shown to have a critical role in cardiac regeneration and can differentiate into EC and promote arteriogenesis [12,13,14]. Figure 5a shows around 2.4% of cells in the IZ were GFP^+^ compared to 0.8% of cells in the NZ, measured by flow cytometry, indicating more GFP^+^ BMSCs homing to CZ for CCG under RI stimulation. Moreover, Figure 5b shows that 1.4% of GFP^+^ cells in the CZ were c-kit positive, whereas only 0.6% of GFP^+^ cells in the NZ were c-kit positive, indicating more c-kit^+^ cells present in the CZ induced by RI. The results show c-kit^+^GFP^+^ BMSCs engrafted more in the CZ than in the NZ of chimeric rat hearts, indicating c-kit^+^GFP^+^ BMSCs contributed to CCG.

### 3.5. BMSCs Contributed to CCG

**Figure 6:**
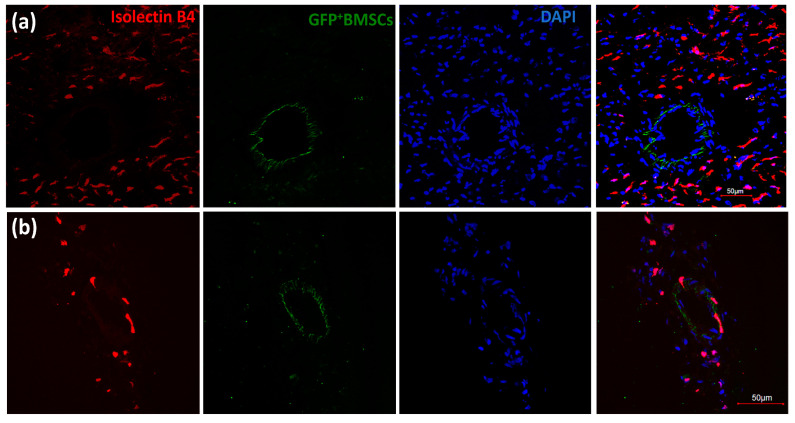
GFP^+^ BMSCs aligned in the walls of large vessels. (**a**,**b**) are confocal images of chimeric rat hearts. The lumen size in (**a**) is bigger than a capillary, suggesting GFP^+^BMSCs contributed to the coronary collateral growth. There is colocalization of GFP^+^BMSCs and isolectin-B4 in (**b**), but the diameter of the vessel is smaller. Green shows GFP^+^ BMSCs. Red shows the EC marker (isolectin-B4). Blue shows nuclei.

Collateral growth differs from angiogenesis because collaterals have coverage of pericytes/smooth muscle cells around the endothelial cell layer, whereas capillaries only have one layer of endothelial cells. Moreover, the size of collaterals is more significant than the size of capillaries, which is smaller than 10 µm. Figure 6 shows GFP^+^ BMSCs aligned around the wall of large vessels bigger than 10 µm. While Figure 6a shows no colocalization of GFP^+^ BMSCs and isolectin-B4 in a vessel with a diameter more significant than 10 µm, Figure 6b shows some colocalization of GFP^+^ BMSCs and isolectin-B4 in a slightly smaller vessel, suggesting that GFP^+^ BMSCs contributed to the CCG induced by RI.

**Figure 7:**
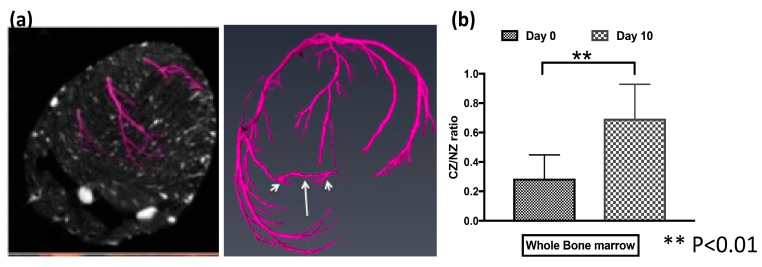
Whole BM cells contributed to coronary collateral growth in the chimeric rats during repetitive ischemia (RI). (**a**) micro-CT images of rat collaterals, marked by arrows. (**b**) The difference in coronary blood flow was measured by contrast echo during inflation of the occluder in the normal zone (NZ) and collateral dependent zone (CZ) at day 0 (before RI) and day 10 (after RI). The CZ/NZ ratio increased after RI, suggesting collateral growth.

To directly confirm the presence of collaterals, we used micro-CT to scan the heart of a chimeric rat after 10 days’ RI. Figure 7a shows a collateral developed between two branches of coronaries during RI. Coronary collaterals can compensate for the low myocardial blood flow during ischemia. Next, we further investigated the collaterals’ function. We measured the coronary blood flow using microspheres or contrast echocardiography in the NZ and CZ of chimeric rats during inflation of the occluder at day 0 (before RI) and day 10 (after RI). We then calculated the CZ/NZ ratio on day 0 and day 10. When the occluder is inflated, the blood flow is decreased in CZ without collaterals, and the ratio of CZ/NZ drops. Figure 7b shows that the CZ/NZ ratio increased after RI, suggesting collateral growth. These structural and functional data show BMSCs contributed to CCG in the chimeric rats during RI.

### 3.6. CD34^+^GFP^+^ BMSCs Are Essential to CCG

**Figure 8:**
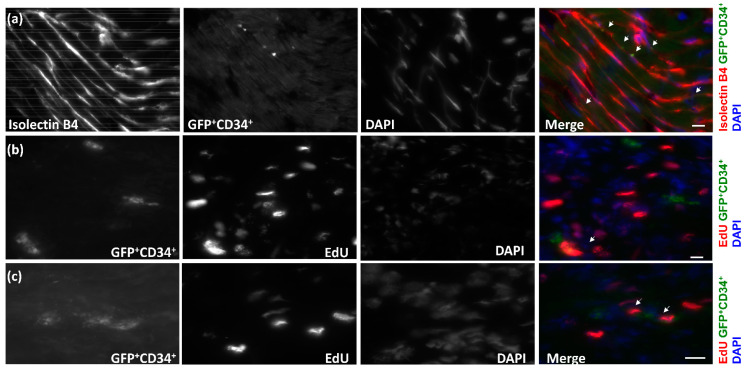
Confocal images of immunostaining of collateral dependent zone (CZ) of GFP^+^CD34^+^ BMT recipient rat heart after 10 days’ repetitive ischemia (RI). (**a**) GFP^+^CD34^+^ BMSCs colocalized with endothelial cell (EC) marker isolectin-B4 (as shown by arrows), indicating that GFP^+^CD34^+^ BMSCs differentiated into EC during coronary collateral growth (CCG) induced by RI. (**b**,**c**) GFP^+^CD34^+^ BMSCs colocalized with EdU (as shown by arrows), suggesting that GFP^+^CD34^+^ BMSCs proliferated during CCG. The scale bar equals 10 μm.

**Figure 9:**
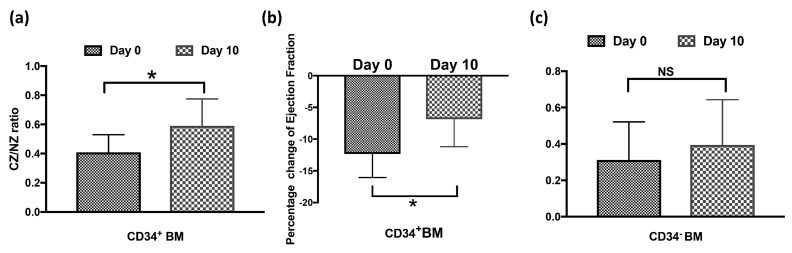
CD34^+^ BMSCs contributed to coronary collateral growth. (**a**) shows the change of coronary blood flow measured by contrast echo during inflation of the occluder in the normal zone (NZ) and collateral dependent zone (CZ) in CD34^+^ BMSC recipient rats at day 0 (before RI) and day 10 (after RI), suggesting coronary collateral growth. * *p* < 0.05 (**b**) shows the ejection fraction change from day 0 to day 10 of RI with occluder inflation, suggesting improved cardiac function and, therefore, coronary collateral growth in CD34^+^ BMSC recipient rats. * *p* < 0.05 (**c**) shows no significant change in CZ/NZ blood flow ratio in CD34^−^ BMSC recipient rats between day 0 and day 10, suggesting no coronary collateral growth. NS = not significant.

After we showed that GFP^+^ BMSCs contributed to the CCG, we further asked which population of bone marrow cells is essential to the process. One population of particular interest is CD34^+^ bone marrow cells, which were shown as one of the markers of endothelial progenitor cells and contributed to angiogenesis or hindlimb collateral growth in animals and humans [15,16,17]. We sorted the CD34^+^GFP^+^ BMSCs or CD34^−^GFP^+^ BMSCs from donor rats and transplanted these populations into the recipient rats. Figure 8a shows that CD34^+^GFP^+^BMSCs presented in CZ of recipient rat hearts and colocalized with isolectin-B4 after 10 days’ RI. To examine whether the CD34^+^GFP^+^ BMSCs proliferated, EdU was injected, as in Figure 4. Figure 8b,c shows GFP^+^CD34^+^ BMSCs colocalized with EdU, suggesting that GFP^+^CD34^+^ BMSCs proliferated during CCG. We also measured the coronary blood flow and cardiac function of the chimeric rats that received CD34^+^ BMSCs. Figure 9a shows that the CZ/NZ ratio increased in CD34^+^ BMSC chimera rats after RI, suggesting collateral growth. We also measured the ejection fraction both before and during inflation of the occluder on day 0 and day 10. Cardiac function decreases during ischemia. Figure 9b shows the ejection fraction change during the inflation of the occluder decreased after RI, suggesting cardiac function improved after RI and further indicating coronary collateral growth occurred in CD34^+^ BMSC recipient rats.

To study whether CD34^+^ BMSCs are essential in this process of CCG, we depleted the CD34^+^ cells from donor bone marrow and transplanted the CD34^−^ BMSCs to the recipient rats; the resulting chimeric rats with CD34^−^ BMSCs survived and went through the RI protocol. Figure 9c shows no significant difference in the ratio of CZ/NZ between day 0 and day 10, indicating no collaterals grew during RI. These results show the essential role of CD34^+^ BMSCs in CCG induced by RI.

## 4. Discussion

This study is the first report on the essential role of BMSCs in a rat model of CCG induced by RI. Our results show that transplanted BMSCs contributed to CCG in the recipient rats. Our findings are summarized as follows. First, the transplanted BMSCs repopulated the recipient rats’ depleted bone marrow. When we used GFP-positive bone marrow cells to trace the fate of the transplanted cells, we found that GFP^+^ BMSCs migrated from the bone marrow to the chimera rat heart’s myocardium under RI stimulation. Interestingly, GFP^+^ BMSCs presented more in the collateral-dependent zone, less in the normal zone, and none in the septum. GFP^+^ BMSCs aligned with the vascular wall of vessels with a diameter more significant than 10µm. These results suggest that hypoxia or shear stress stimulated the GFP^+^ BMSCs’ homing to the CZ during the RI.

Second, GFP^+^ BMSCs in the CZ proliferated, shown by colocalization with EdU at the early stages of CCG (day 3–5 of RI protocol), and differentiated into endothelial cells, demonstrated by colocalization with EC marker isolectin-B4. In addition, more c-kit^+^GFP^+^ BMSCs were present in the CZ than the NZ in chimeric rat hearts, suggesting the subpopulation of c-kit^+^ BMSCs participated in the collateral growth. It has been reported that c-kit signaling is essential to arteriogenesis in hindlimb ischemia, and c-kit^+^ cells play a critical role in cardiac regeneration [12,13,14]. We do not know whether these c-kit^+^ BMSCs differentiated into EC for blood vessel growth or if they had a paracrine effect of stimulating collateral growth. Future studies will warrant addressing these questions.

Third, this study further shows that GFP^+^ BMSCs contributed to collateral growth. We mentioned the colocalization of GFP^+^ BMSCs with isolectin-B4, which could be angiogenesis or arteriogenesis. However, large GFP^+^ vessels and micro-CT images of coronary collaterals indicated that GFP^+^ BMSCs specifically contributed to arteriogenesis/collateral growth. Moreover, the study shows the functional consequences of coronary collaterals in the recipients, such as improving coronary blood flow and cardiac function during ischemia. The functional data from transplanted GFP^+^ BMSCs indicate that GFP^+^ BMSCs play an essential role in CCG. This aspect is critical for the outcome and implications of stem cell transplantation and therapy.

Fourth, this study shows that CD34^+^ BMSCs are essential to CCG. CD34 is one of the endothelial progenitor cell markers, and CD34^+^ cells have been used in cell therapy for regenerating endothelial cells and promoting angiogenesis in cardiovascular diseases [15,16,17,18]. Although CD34^+^ BMSCs and CD34^−^ BMSCs both rescued the recipient rats from death, and blood analysis of recipients did not show any significant changes among the chimeras receiving CD34^+^ BMSCs, CD34^−^ BMSCs and whole BMSCs (data not shown), however, coronary collaterals grew only in the CD34^+^ BMSC recipient chimeras; no collateral developed in CD34^−^ BMSC recipient chimeras. CD34^+^ BMSCs proliferated and differentiated into endothelial cells in the CZ for collateral growth. The depletion of CD34^+^ cells in bone marrow led to impaired CCG induced by RI. This study indicates the essential role of CD34^+^ BMSCs in CCG.

We are aware of the injury caused by lethal irradiation. While we alternately tried to deplete the recipients’ bone marrow by withdrawing the maximum volume of bone marrow before injecting the donor bone marrow cells, the uncertainty of the extent of depletion of the recipient’s bone marrow limited us in drawing a solid conclusion. It would be interesting to do bone marrow transplantation in other animal models of CCG, such as hypoxia or distant ligation of LAD [19,20], to confirm our findings in this study.

Our previous study showed that endothelial sprouting is essential in CCG induced by RI, CXCR4, hypoxia-Hif1⍺, VEGF-VEGFR2, JAG1, and MCP-1 signaling are critical [8]. Bone marrow-derived endothelial progenitor cells might contribute to endothelial sprouting during CCG. EPCs were reported to correlate with revascularization and improve cardiac function in patients with coronary chronic total occlusions [21]. Furthermore, immune cells in bone marrow might regulate the immune response to hypoxia and inflammation during RI. In a rabbit model of chronic limb ischemia, bone marrow mononuclear cells improved collateral circulation after transplantation [22].

Moreover, the paracrine effect of BMSCs might contribute to CCG when homed and engrafted in the CZ of the myocardium. A limitation of this study is the lack of a mechanism to explain how bone marrow cells are homing to the CZ to promote collateral growth and why CD34^+^ BMSCs are essential to coronary collateral growth. Single-cell RNA sequencing of GFP^+^ BMSCs and CD34^+^ BMSCs from the recipient rat hearts at different stages of CCG or spatial sequencing of CZ and NZ of GFP^+^ BMSCs or CD34^+^ BMSCs recipients will be helpful to address these questions.

Coronary collaterals have great potential for treating IHD patients. This study shows endogenous bone marrow stem cells contributed to coronary collateral growth, and CD34^+^ BMSCs are essential to CCG. The results point to a potential therapeutic approach to regulate bone marrow homing and function to promote CCG in IHD where BMSCs are dysfunctional under oxidative stress from risk factors such as diabetes, hyperlipidemia, and hyperglycemia [23,24]. microRNA has been targeted to regulate BMSC dysfunction caused by oxidative stress in IHD [24,25,26]. Although microRNA is associated with coronary collateral artery function in chronic total occlusion patients [27], microRNA regulation has not yet been used to induce coronary collateral growth. It would be worthwhile exploring this area in future studies.

## Data Availability

The data supporting this study’s findings are available from the corresponding author upon reasonable request.
